# Devastating dengue outbreak amidst COVID-19 pandemic in Bangladesh: an alarming situation

**DOI:** 10.1186/s41182-022-00401-y

**Published:** 2022-01-25

**Authors:** Mohammad Mehedi Hasan, Abdul Moiz Sahito, Muhammad Muzzamil, Parvathy Mohanan, Zarmina Islam, Md. Masum Billah, Mohammod Johirul Islam, Mohammad Yasir Essar

**Affiliations:** 1 Department of Biochemistry and Molecular Biology, Faculty of Life Science, Mawlana Bhashani Science and Technology University, Tangail, Bangladesh; 2 Dow University of Health Sciences, Karachi, Pakistan; 3 Health Services Academy, Islamabad, Pakistan; 4 Department of General Medicine, Medical University Sofia, Sofia, Bulgaria; 5 Department of Pharmacy, Faculty of Life Science, Mawlana Bhashani Science and Technology University, Tangail, Bangladesh; 6 Kabul University of Medical Sciences, Kabul, 1001 Afghanistan

**Keywords:** Dengue fever, COVID-19, Challenges, Public health, Bangladesh

## Abstract

Dengue fever is a viral infection caused by *Aedes* mosquitoes that has recently expanded fast in many of the WHO member states globally. Female mosquitoes, mostly *Aedes aegypti* and, to a smaller degree, *Aedes albopictus*, disseminate dengue virus. Dengue fever has been more common in recent decades all across the world, and Bangladesh is no exception. As the COVID-19 outbreak wreaks havoc, the following rise in dengue illnesses has been a source of considerable concern. As the health care has been stretched thin in these dangerous times, the vulnerable population has been left at the mercy of these two viral infections. Lack of knowledge, major legislative changes, poor eradication initiatives, and a lack of financing resources have all contributed to the increase in numbers. Stakeholders and policymakers must begin taking meaningful actions and implementing well-thought-out adjustments immediately, or the situation will worsen, resulting in the loss of thousands of innocent lives.

## Introduction

Dengue fever is a viral infection caused by mosquitoes that has recently expanded rapidly in recent years throughout all WHO member states [1]. Dengue virus (DENV) has four separate but highly correlated genetic variants (DENV 1–4) [1]. Dengue fever occurs throughout the tropics, with local variations in risk influenced by variables such as relative humidity, temperature, rainfall, and unplanned rapid urbanization. Dengue fever affects human health and also worldwide economies [2, 3]. As Bangladesh grapples with the threat of a resurgent coronavirus, an alarming spike in seasonal dengue disease has presented officials with a new challenge. However, in 2021, when the majority of health resources, such as human resource, laboratory testing, hospitals, and epidemiological monitoring, are dedicated to COVID-19, other illnesses, like dengue fever, suffer a substantial delay regarding diagnosis and treatment. Furthermore, during the COVID-19 pandemic, healthcare practitioners in dengue-endemic regions or treating patients who have recently been to these areas must include dengue and COVID-19 in the differential diagnosis of acute febrile diseases. Clinical management for persons who acquire severe sickness from any of these two disorders is considerably different, frequently necessitating hospital-based care, putting further load on an already crippled system.

Dengue fever has become more widespread in past few decades everywhere in the world. Based one projection, more than 380 million people are infected with DENV annually, including 95 million manifesting clinically (with any severity of disease) [4]. Bangladesh's situation is no exception. Authorities in Bangladesh recorded 1,405 cases of dengue fever in 2020, 101,354 cases in 2019, and 10,148 cases in 2018 [5, 6]. Between September 15 and October 4, 2021, the Directorate General of Health Services (DGHS) documented another 4,624 instances of dengue disease, amounting this year to 19,133 [6]. This worrisome spike in case counts is explained in part by a shift in national policies for recording and reporting dengue fever to Ministries of Health (MoH) and the world health organization (WHO). The International Health Regulations (IHR) compel governments to make frequent and particular efforts to improve resistance to such epidemic-prone diseases. Such activities necessitate investments in monitoring and preparedness capability for early disease outbreak detection and reporting, as well as effective and prompt public health actions for confinement and alleviation, encompassing cross-border and global information exchange and coordination. As a consequence, national commitment to controlling the disease is in line with the aims and criteria of the IHR [7].

However, it also reflects the government's acknowledgment of the burden, and therefore the need of reporting dengue illness burden. As a result, while the whole global burden of the illness is unknown, the observed rise only gets us closer to a more precise assessment of the full extent of the burden [1]. The COVID-19 pandemic is putting a huge strain on global healthcare and management systems [8, 9]. It is extremely alarming that numerous other infectious illnesses have been on the increase concurrently with the COVID-19 epidemic, causing havoc on the world healthcare system [10, 11].

At this critical juncture, as case numbers grow in a number of countries, exposing individuals to the highest risk for both illnesses, the WHO has emphasized the need of ongoing actions to avert, identify, and address vector-borne diseases such as dengue and other arboviral infections. The collective consequences of the dengue and COVID-19 epidemics has the potential to be catastrophic for individuals who are vulnerable. Similarly, as the COVID-19 situation unfolds, Bangladesh's healthcare industry is under jeopardy. During this critical moment, when the country's healthcare system is at maximum capacity, an added threat of dengue outbreaks may have a domino effect, bringing the entire system down. Bangladesh must create clear priorities to address each of these issues before the situation deteriorates. The purpose of this article is to evaluate the present state of dengue in Bangladesh in order to better understand the existing efforts and difficulties, as well as to find possibilities for development through innovative suggestions for all stakeholders.

## Challenges and efforts

Bangladesh's healthcare system was already in disarray owing to a devastating third wave of the coronavirus. It was exacerbated further by an increase in dengue incidence, resulting in a double illness load on the healthcare system [12]. The same symptoms and false-positive test results for COVID-19 in dengue patients make it difficult for practitioners to recognize between dengue and COVID-19. Furthermore, due to the wide array of disease indications and the absence of clear case definitions between COVID-19 and dengue, many instances of dengue went unreported. The clinical presentation of both the diseases are similar in symptoms like fever, muscle or body ache, nausea, vomiting, and others leading to a high possibility for misdiagnosis [13]. Furthermore, in individuals who present with co-infection with both dengue and COVID-19 at the same time, it may result in death or leave the patient in a vulnerable health situation.

Bangladesh has less than 2000 ventilators for a population of more than 16 million people, implying that one ventilator is accessible for every 93,273 people. Furthermore, practically all hospitals are located in metropolitan regions, making these well-equipped institutions inaccessible to rural people [14]. Because of the suddenness of this epidemic, Bangladesh's situation is extremely vulnerable. It has been observed that a lack of preparedness, including weak public health infrastructure, ineffective vector-control measures, and a frail healthcare system, leads to catastrophic DENV outbreaks [15]. Expeditious advancement of COVID-19 caused paucity of testing kits, protective equipment’s and this caused a lack of responsiveness in so many situations following a spike in dengue cases [14]. All of these circumstances exist in Bangladesh, making it a hotspot for the emergence of a severe dengue threat. As the hospitals were already burdened with the people who were affected with COVID-19 virus. Dengue cases double the burden amid pandemics. Over 15,000 dengue illnesses and approximately 57 deaths have been reported in Bangladesh since January 2021 [16]. According to the DGHS, Dhaka accounted for 88% of the overall cases since January. DENV-3 was described as the dominant serotype in this year 2021 outbreak. DENV-3 is more type that is resistant and chances of mortality are higher than other type due to virus’s characteristic antibody-dependent enhancement [17]. Initially the rise in the number of dengue cases were not getting any required attention due to COVID-19, but it was addressed when the health care system was further burdened and dengue-infected cases were unattained. Moreover, the fragile health care system was labeled as a disastrous failure due to improper distribution of hospital beds, diagnostic kits, and an increase in the number of deaths due to dengue of people and health care professionals.

The city corporation authorities are taking measures via vector control, which is eliminating mosquito breeding places, as the number of cases increased. Encouragement of community knowledge is a preventative measure and control activity. In addition to these efforts, home changes such as wearing full-sleeved protective clothes, applying insect repellents, sleeping under mosquito nets at night, and removing sources of standing water can decrease the potential for infected mosquitoes to breed near human populations.

## Public health implications

Dengue epidemics have been known to ruin the healthcare system by overwhelming it with a significant number of patients. During a pandemic, healthcare facilities struggle to find adequate space to care for all arriving patients, thus patients are routinely transferred between locations or sent to centers farther distant from their residences that are more suited to meet the need [18]. During a dengue outbreak, a surge in the number of patients has a serious influence on inpatient admissions and long waits in the hospital. According to reports, the rapid influx of patients resulted in delayed diagnosis of dengue and other infections. Bangladesh, a lower-middle-income country [19], is confronted with all of these difficulties, since its meager healthcare resources have already been drained as a result of COVID-19. As a result, the public's health is jeopardized in a catastrophic way. It has also been demonstrated that the quantity of patients seen throughout an epidemic contributes to the total expense of disease management at the hospital level [18]. According to healthcare practitioners who have worked in both the public and commercial sectors, the public sector offered more significant patient management challenges. According to a research done in Brazil, self-medication rates were high during the outset of a dengue outbreak [18]. As symptoms increased, patients would migrate from self-medication to clinics.

## Recommendation

A recent study showed that many people in Bangladesh still do not have the fundamental information on dengue. There are still a few misunderstandings about where the dengue vector breeds. The most typical breeding locations for dengue mosquitoes, according to many, are filthy and polluted water, such as sewage channels, which is a massive misconception [20]. For effective dengue management initiatives, public education on the disease should be prioritized. Effective educational events, public health initiatives by non-governmental organizations (NGOs), and the MoH should be conducted particularly about dengue transmission. Early recognition of the *Aedes* mosquito's breeding sites, early diagnosis, and therapeutic methods are extremely important. The MoH should supply adequate health personnel and train them to provide appropriate counseling in order to promote changes in behavior among the populace in order to advance dengue prevention. Additionally, constant awareness and surveillance must be carried out to ensure long-term dengue prevention practices.

Cross-reactivity between antibodies to DENV and COVID-19 present the need for a diagnostic tool that can differentiate the two and hence, double laboratory tests may help. However, beyond this, low diagnostic and healthcare capacity remains a major concern for Bangladesh, so increasing both the number and convenience of location of laboratories can help.

Policy-makers can work on establishing an app that identifies high-risk areas for dengue, and provide a symptomatic quiz that can be used to self-assess one’s own symptoms. The quiz can be utilized to recommend diagnostic tests based on primary symptoms for dengue or COVID-19 such as PCR or antigen test. A reliable self-assessment exam based on symptoms, coupled with a heat map of affected areas can guide citizens to equip themselves with appropriate protection [21].

To address this further, the negative PCR and antigen test upon arrival to Bangladesh should be made mandatory for inbound flights to curb the spread of new variants of COVID-19. Lastly, policies should work towards implementing telemedicine solutions to areas that do not have access to diagnostic facilities or appropriate treatment. This can help further identify areas that may not have appropriate epidemiological surveillance. Policies should also focus on strict measures for social distancing, educational programs for both parents and children, and availability of both dengue and COVID testing services in educational institution [22].

In rural and urban areas, public education campaigns to educate people regarding dengue transmission, diagnosis, and treatment is necessary and can be employed through local visits by community healthcare workers, radio broadcast with religious leaders and healthcare professionals (HCPs) to encourage the use of preventive methods, and TV as well as Facebook advertisements to better inform those in urban areas. Combating disease-carrying mosquitos, particularly *Aedes aegypti* and *Aedes albopictus*, is the most effective strategy to prevent DENV transmission. It is well accepted that sleeping under a bed net can help avoid these dengue vectors [23]. Because these nets are widely available, easily accessible, and simple to use, they are one of the most effective and feasible methods of preventing dengue. Authorities should ensure that residents in high-risk locations have access to these nets. Counseling services, workshops, and training for both medical students and HCPs regarding dengue is crucial, especially in a diagnostic and clinical capacity to differentiate the two. NGOs should work on spraying campaigns in rural areas with high risk to reduce transmission. Symptoms like mucosal bleeding, stomach pain, vomiting, fluid accumulation, and drowsiness should be given the highest priority in terms of management. In each of the influenced regions, efforts must be taken for differential diagnosis in laboratories. Required medications, gears, and testing labs should be given to minimal health care units for reinforcing the healthcare framework to get ready for the overwhelming cases of dengue fever with the progressing burden of COVID-19 patients. The healthcare workers should embrace preparatory measures for themselves and be made mindful of the complexity created by dengue fever and COVID-19. There must be strict contamination prevention and control measures in all health care levels to guarantee adequate clinical care to dengue cases during the pandemic. The circulation of guidelines from the regulatory board to primary healthcare units might help the early differentiation of both cases as well as decrease severe consequences. Failure to set up and execute appropriate policies might lead to the dengue outbreak with the burdens of the simultaneous COVID pandemic, resulting in the collapse of the health and social framework, as well as the economic development of the nation.

## Conclusion

In conclusion, Bangladesh, with a concurrent COVID-19 and dengue outbreak has been severely impacted because of low healthcare capacity, poor surveillance, and additional socioeconomic disparities. Present challenges including population density in urban centers, a monsoon season promoting breeding sites, weak healthcare infrastructure, a history of dengue outbreaks, COVID-19 and dengue cross-reactivity as well as symptom similarity have complicated predisposing conditions. Hence, efforts at unique diagnostic testing, epidemiological surveillance, educational campaigns are needed to effectively mitigate this crisis.

## Data Availability

Not applicable.

## Abbreviations

COVID-19: Coronavirus disease 2019
DENV: Dengue virus
WHO: World Health Organization
MoH: Ministries of Health
DGHS: Directorate General of Health Services
NGOs: Non-governmental organizations
HCPs: Healthcare professionals
IHR: International Health Regulations
